# An Update on the Effects of Vitamin D on the Immune System and Autoimmune Diseases

**DOI:** 10.3390/ijms23179784

**Published:** 2022-08-29

**Authors:** Claudia Sîrbe, Simona Rednic, Alina Grama, Tudor Lucian Pop

**Affiliations:** 1 2nd Pediatric Discipline, Department of Mother and Child, “Iuliu Hatieganu” University of Medicine and Pharmacy, 400012 Cluj-Napoca, Romania; 2 2nd Pediatric Clinic, Emergency Clinical Hospital for Children, 400177 Cluj-Napoca, Romania; 3 Rheumatology Department, Emergency County Hospital Cluj, 400347 Cluj-Napoca, Romania; 4 Rheumatology Discipline, “Iuliu Hatieganu” University of Medicine and Pharmacy, 400012 Cluj-Napoca, Romania

**Keywords:** vitamin D, immune function, immunomodulation, immune-related diseases, autoimmune disorders, monocytes, macrophages, inflammation

## Abstract

Vitamin D intervenes in calcium and phosphate metabolism and bone homeostasis. Experimental studies have shown that 1,25-dihydroxyvitamin D (calcitriol) generates immunologic activities on the innate and adaptive immune system and endothelial membrane stability. Low levels of serum 25-hydroxyvitamin D (25(OH)D) are associated with an increased risk of developing immune-related diseases such as psoriasis, type 1 diabetes, multiple sclerosis, and autoimmune diseases. Various clinical trials describe the efficacy of supplementation of vitamin D and its metabolites for treating these diseases that result in variable outcomes. Different disease outcomes are observed in treatment with vitamin D as high inter-individual difference is present with complex gene expression in human peripheral blood mononuclear cells. However, it is still not fully known what level of serum 25(OH)D is needed. The current recommendation is to increase vitamin D intake and have enough sunlight exposure to have serum 25(OH)D at a level of 30 ng/mL (75 nmol/L) and better at 40–60 ng/mL (100–150 nmol/L) to obtain the optimal health benefits of vitamin D.

## 1. Introduction

The role of vitamin D in health was first defined by its deficiency that results in rickets in children and osteomalacia in adults [1]. Vitamin D was first described in the early 1600s [2], and despite its name, it is a prohormone because humans do not obtain it only from their diet. Vitamin D is produced after exposure to ultraviolet B radiation (wavelength 290–315 nm) and can also be obtained from diet and supplements [3].

Vitamin D (calciferol) has two forms D2 (ergocalciferol) and D3 (cholecalciferol) [4]. After exposure to ultraviolet B radiation, cholecalciferol is produced in the skin from the precursor protein 7-dehydrocholesterol. The main supply of vitamin D derives from the skin; the diet only contributes in a small amount. The proteins involved in the transport of vitamin D are albumin and lipoproteins, most of vitamin D being transported in an inactive form by D-binding protein (DBP) to the liver. DBP also has an immunomodulatory role and intervenes in bone development [1,3,4,5]. In the liver, vitamin D is converted to 25-hydroxyvitamin D (25(OH)D), an intermediate inactive form in the presence of 25-hydroxylase encoded by the CYP2R1 allele [6]. The 25(OH)D is then transported by DBP to the kidney and converted to the active form, also called calcitriol, in the presence of 1α-hydroxylase encoded by the CYP27B1 allele [6]. This enzyme can be found in various cell types (i.e., skin, bone cells, immune cells, placenta), but the highest concentration is expressed in the proximal tubule in the kidney [1,3,4,5]. In the kidney, hydroxylation is controlled by parathyroid hormone (PTH), calcium and phosphate levels [7]. After passing through the cell membrane, 1,25-hydroxyvitamin D forms the 1,25-hydroxyvitamin D—vitamin D receptor (VDR) complex that modulates gene expression in the nucleus [7]. The outcome of this interaction is calcium homeostasis which inflects intestinal calcium absorption. In the case of low 1,25-hydroxyvitamin D levels, calcium will be absorbed from the bone in favor of the intestine [8]. Breakdown of both 25(OH)D and 1,25-hydroxyvitamin D is performed by the same 24-hydroxylation enzyme (CYP24A1) [1,3,4,5].

There are alternative pathways of vitamin D activation different from 25(OH)D and 1,25-hydroxyvitamin D. This novel in vivo pathway of vitamin D metabolism initiated by P450sc includes: 20-hydroxyvitamin D3 [20(OH)D3], 22(OH)D3, 20,23(OH)2D3, 20,22(OH)2D3, 1,20(OH)2D3, 1,20,23(OH)3D3, and 17,20,23(OH)3D3. These novel metabolites are produced by the placenta, adrenal glands and, at low levels, by epidermal keratinocytes. The predominant human in vivo production of 20(OH)D3 is approximately 20 times more reduced than 25(OH)D. The role of cytochrome P450scc (CYP11A1) and CYP27B1 enzymes in vitamin D metabolism was demonstrated in studies using isolated mitochondria and purified enzymes [9,10]. In addition, CYP11A1-derived secosteroids produced in vivo in the skin, serum and adrenal gland manifest their biological activity as hormones [11]. In the placenta and adrenal glands, the main pathway comprised 20(OH)D3, 20,23(OH)2D3, 17,20,23(OH)3D3, and minor pathways included 25(OH)D and 1,25(OH)2D, and 22(OH)D3 and 20,22(OH)2D3. In epidermal keratinocytes, the predominant metabolites were 22(OH)D3 and 20,22(OH)2D3 [9].

Similarly, human placentas, rat and bovine adrenal glands, human epidermal keratinocytes, and colon cancer Caco-2 cells can produce alternative pathways of ergocalciferol. The novel hydroxy-derivatives include 20(OH)D2, 17,20(OH)2D2, 1,20(OH)2D2, 25(OH)D2 and 1,25(OH)2D2 with high CYP11A1 and CYP27B1 expression. These pathway products present tissue- and cell-type specificity with a higher production of 25(OH)D2 in placentas and Caco-2 cells, and 20(OH)D2 and 25(OH)D2 production in human keratinocytes [12].

CYP11A1 can convert vitamin D into the non-calcemic analog 20S-hydroxyvitamin D3 [20S(OH)D3]. This analog could be used as a potential treatment for rheumatoid arthritis (RA) and other autoimmune disorders by suppressing clinical signs of arthritis and repairing joint damage in a mouse model. 20S(OH)D3 can also decrease the number of CD4 and CD19 cells, reducing inflammatory cytokines. Arthritis can be attenuated by 20S(OH)D3 by reducing pro-inflammatory cytokines and antibodies against type II collagen [13].

Alternative nuclear receptors for CYP11A1-derived vitamin D-hydroxy-derivatives comprise the retinoid-related orphan receptors (ROR)α and γ [14], the arylhydrocarbon receptor (AhR) [15], and liver X receptors α and β [16,17]. Notably, CYP11A1 is expressed in the immune system, in human [18] and murine [19] T cells, in human CD4, CD8, B cells and monocytes [20]. CYP11A1 is involved in the local production of steroidogenesis in the skin and the specific vitamin D metabolism, lumisterol, and 7-dehydrocholesterol. CYP11A1 metabolites play a role in the protective barrier and skin immune functions. Malfunction of CYP11A1 can result in skin disorders [21]. CYP11A1 hydroxy-derivatives and lumisterol hydroxy-derivatives present photoprotective effects by stimulating intracellular free radical scavenging and DNA repair. As photoprotective agents in human keratinocytes, CYP11A1 hydroxy-derivatives are involved in p53-phosphorylation, in the antioxidant response regulated by NRF2, and induction of DNA repair [17].

Human epidermis and serum production of a photoproduct of pre-vitamin D, CYP11A1 and CYP27A1 hydroxylate tachysterol3 produce 20S-hydroxytachysterol3 [20S(OH)T3] and 25(OH)T3. These metabolites can inhibit the proliferation of epidermal keratinocytes and dermal fibroblasts similar to 1,25-dihydroxyvitamin D resulting in stimulation of the differentiation and anti-oxidative genes in keratinocytes. They attach to VDR, express CYP24A1, activate AhR, and bind to the ligan-binding domain (LBD) of LXR α and β, and the peroxisome proliferator-activated receptor γ (PPARγ). The biological function of tachysterol3 is demonstrated by endogenous production of 20S(OH)T3 and 25(OH)T3, which can interact with VDR, AhR, LXRs, and PPARγ [22].

VDR is associated with the nuclear receptor superfamily. Ligand-receptor dimerization is established with the retinoic X receptor (RXR). This complex attaches to the vitamin D responsive elements (VDREs), inducing gene expression by targeting promoter sequences of calcitriol, underlining its genomic function [23,24,25]. The non-genomic effect of vitamin D is highlighted by the connection of calcitriol to a membrane-bound to VDR and caveolin-1 [26].

After activation, vitamin D binds to calcium transporting proteins in the small intestine triggering calcium absorption [26,27]. Vitamin D stimulates osteoclasts leading to bone resorption and increased blood calcium [28]. In bone growth, vitamin D supports collagen matrices and osteoblasts mineralization [29]. Vitamin D induces the expression of osteocalcin, an important non-collagenous bone protein, and stimulates bone resorption RANKL (receptor activator of nuclear factor kappaB ligand), a TNF family member [3,30,31,32]. Vitamin D with parathyroid hormone (PTH) and fibroblast growth factor 23 (FGF23) regulate calcium and phosphate homeostasis [33]. The negative feedback consists of vitamin D directly inhibiting PTH production, which decreases bone resorption and increases urinary calcium excretion. In this loop, osteocytes induce FGF23 production, increasing urinary phosphate excretion [30,33,34].

The clinically significant reserves of vitamin D are serum 25(OH)D levels (defined as the sum of ergocalciferol and 25(OH)D). In contrast, calcitriol has a short half-life (4–8 h) and depends on calcium homeostasis. An important debate is the proper standardization of tests [35]. Two different methodologies are used: competitive immunoassays (radio-immunoassays or binding-protein assays) and procedures that use high-performance liquid chromatography and liquid chromatography tandem-mass spectrometry. The latter methods are the gold standard procedures [28,36,37].

The Clinical Guidelines Subcommittee of The Endocrine Society defines the deficiency of 25(OH)D to be less than 20 ng/mL (50 nmol/L) [38], while the Institute of Medicine (US) Committee to Review Dietary Reference Intakes for Vitamin D and Calcium defined it to be less than 12 ng/mL (30 nmol/L) [39]. The first society defined insufficiency at levels of 21–29 ng/mL (52 to 72 nmol/L) [38], while the latter at levels of 12–20 ng/mL (30–50 nmol/L) [39]. Vitamin D intoxication is described at values higher than 150 ng/mL (374 nmol/L) [3].

Considering these values, a study based on 6275 American children and adolescents revealed that 61% presented 25(OH)D insufficiency and 9% deficiency [40]. A high prevalence (up to 40%) of vitamin D insufficiency and 6% deficiency is described in adults [41,42,43]. The first internationally standardized dataset of vitamin D status across a latitude gradient of 35° N to 69° N that enrolled almost 56,000 individuals reported that 13% of EU participants had serum 25(OH)D < 30 nmol/L and 40% presented levels < 50 nmol/L [44,45]. Although a trend is described toward lower 25(OH)D levels in the population [45], the current recommendation is not population-wide screening for vitamin D deficiency but testing the individuals that are at risk of developing vitamin D deficiency, such as patients suffering from hyper- and hypoparathyroidism, osteoporosis, and kidney disease [38,46,47,48].

There is a direct relationship between the dose of vitamin D and serum 25(OH)D with a linear curve at a daily dose of 1000–2000 IU (25–50 μg), but this curve flattens at higher doses [48]. A daily intake of 1040 IU (26 μg) in vitamin D deficiency and 400 IU (10 μg) in vitamin D insufficiency is needed to obtain a concentration above 20 ng/mL (50 nmol/L) in 97.5% of the population [49]. Several studies have tried finding the optimal vitamin D dose to correct the deficiency. These studies concluded that most substitutions resulted in adequate 25(OH)D levels regardless of the interval or dosing regimen. Higher loading regimen doses result in a faster insufficiency correction [50,51,52,53,54,55,56,57,58]. Once the values of vitamin D are corrected, the Endocrine Society recommends a daily dose of 600–2000 IU (correction depending on the exposure to sunlight), and IOM recommends a daily dose of 600 IU [38,39,59]. Daily exposure of 7–30 min to sunlight is estimated to be enough to accomplish vitamin D substitution doses considering the skin color, season, and latitude [59]. Vitamin D substitution in adults aged 70 years or older without major comorbidities did not lead to significant differences in improvement in blood pressure, physical performance, fractures, infection rates, or cognitive function [60,61]. One nationwide, randomized, controlled trial including 25,871 participants, proved that vitamin D supplementation for five years, with or without omega 3 fatty acids, can decrease the risk of autoimmune disease by 22%. Omega 3 fatty acid supplementation with or without vitamin D can decrease the risk of autoimmune disease by 15% [62]. Hypercalciuria can be more frequently encountered with higher doses. In a double-blind, randomized controlled trial with 373 participants, hypercalcemia was rare, mild, and transient at higher doses. The safety of vitamin D supplementation is comparable for doses of 400, 4000, and 10,000 IU/day [61,63].

Food-based solutions for optimal vitamin D nutrition and health through the life cycle (European Commission funded project ODIN) was a cross-disciplinary project that included 30 partners from 19 countries, which aimed to prevent vitamin D deficiency and develop a public health food-first approach [64,65]. To address the problem of dietary requirements for vitamin D, four Randomized Controlled Trials (RCTs) [66,67,68,69,70] were conducted in Northern Europe to obtain 25(OH)D targets of 25/30 to 50 nmol/L in almost all participants with absent or reduced sun exposure. One RCT revealed that vitamin D intake of 8 and 13 μg/day can prevent vitamin D deficiency in white children and adolescents [66,67]. One meta-analysis of RCTs evaluating the use of vitamin D-fortified food among healthy children aged 1–18 years old reported that vitamin D food fortification prevented vitamin D deficiency and improved IQ levels [71]. White women during pregnancy require 30 μg/day to maintain serum 25(OH)D levels ≥50 nmol/L during pregnancy to obtain 25(OH)D concentration of >25 nmol/L in 99% and ≥30 nmol/L in 95% of umbilical cord serum [70]. Among women of ethnic minorities in Northern Europe, white women in Finland required 8 μg/day to obtain the cut-off value of 30 nmol/L, while women of East African descent needed 18 μg/day during wintertime [69]. ODIN research demonstrated that diverse fortification strategies could prevent vitamin D deficiency in population subgroups [64,65]. Finland has a mandatory fortification program of milk products with a positive impact on vitamin D nutrition [68].

Genetic and environmental factors have been known to influence the appearance of autoimmune diseases [72]. Among the well-known function of vitamin D in supporting bone mineralization through calcium homeostasis [73], vitamin D has been demonstrated to intervene in innate [74] and adaptive [75] immunity by controlling the growth and differentiation of different immune cells such as T [76] and B lymphocytes [77], macrophages [78] and dendritic cells [79]. Vitamin D is associated with systemic [80] or organ-specific [81] autoimmune diseases.

Beyond its classical role in calcium homeostasis, evidence arose from studies demonstrating antioxidant [82,83,84] and anti-fibrotic [85,86,87] functions, preventing inflammatory response [88] and intervening in immune-mediated injuries [3,8,89,90,91,92,93,94]. Treatment opportunities influence the prognosis and quality of life in patients with autoimmune diseases, and the immunomodulatory effects of vitamin D represent a potential supplementation therapy as vitamin D is deficient in patients suffering from autoimmune diseases [29].

Vitamin D has pleiotropic effects suggested by the expression of VDR in lymphocytes and dendritic cells. Many studies are based on vitamin D’s role in various diseases such as autoimmune disorders, cardiovascular diseases and tumors. In autoimmune and inflammatory diseases, vitamin D intervenes in the innate and adaptive immune systems [3,8,83,85,88,91,92,93,94]. Animal studies have demonstrated that administering vitamin D or its analogs can influence the occurrence and progression of many immune-related disorders [5,29]. This underlines that vitamin D can lead to changes in the incidence and severity of various diseases such as infectious diseases, psoriasis, rheumatoid arthritis, type 1 diabetes, and multiple sclerosis [95]. Therefore, this narrative review discusses the effects of vitamin D on the immune system and the role of vitamin D in the pathogenesis of various immune-mediated and autoimmune diseases.

## 2. Vitamin D Effects on the Innate Immune System

**Figure 1:**
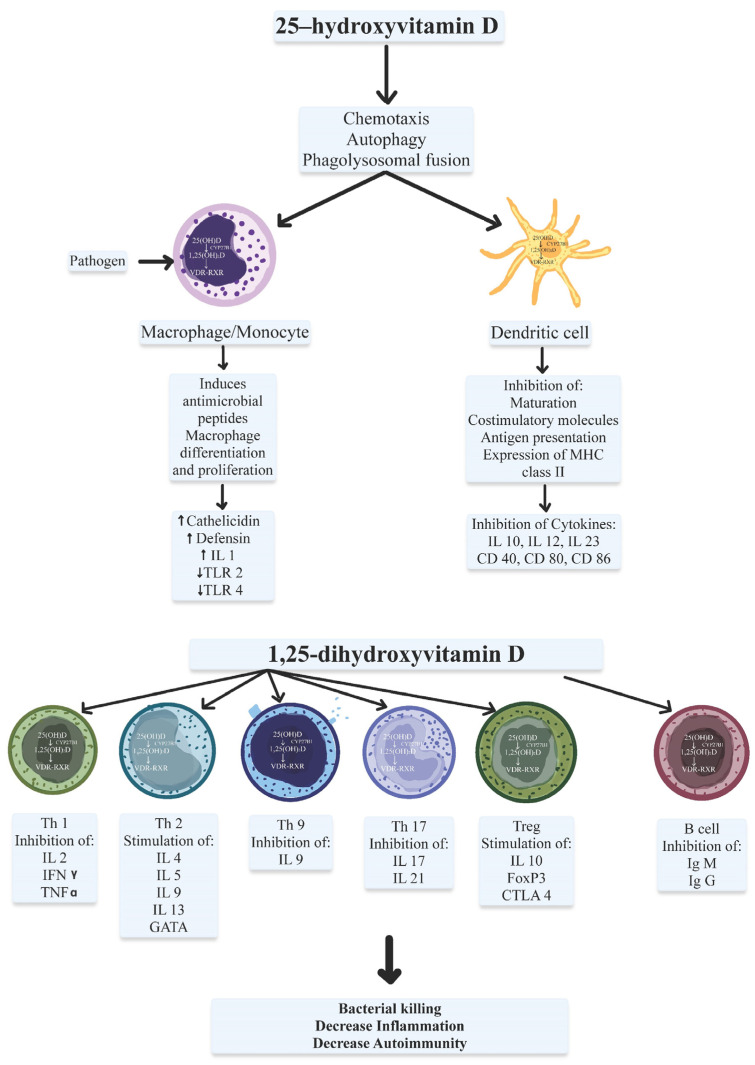
Immunomodulatory actions of active vitamin D (1,25-dihydroxyvitamin D3; 1,25-(OH)2D3) include effects on the innate and adaptative immune systems. 1,25-(OH)2D3 exerts its effect via direct binding on both VDR on the dendritic cell (DC) and the T lymphocytes. Calcitriol intervenes in APC differentiation and function by promoting APC to become more tolerogenic and decreasing the expression of major histocompatibility complex (MHC) class II and other similar molecules on the cell surface. Vitamin D may also reduce T helper (Th) cell differentiation and proliferation and induce a more tolerogenic immune response than a pro-inflammatory status with induction of T helper-2 (Th 2)-lymphocytes and regulatory T lymphocytes (Tregs), with downregulation of the pro-inflammatory T helper-1 (Th 1) lymphocytes, T helper-17 (Th 17) lymphocytes, and T helper-9 (Th 9) lymphocytes. Other abbreviations: IL: interleukin; Ig M: immunoglobulin M; Ig G: immunoglobulin G; IFN-γ: interferon-γ; TNF-α: tumor necrosis factor-α; toll-like receptors (TLRs), GATA-3: GATA binding protein-3; FoxP3: forkhead box P3, CTLA-4: cytotoxic T lymphocyte-associated protein-4.

Calcitriol is a pluripotent regulator of the innate immune system (Figure 1). The bacterial infection triggers the activation of toll-like receptors (TLRs) that regulates VDRs expression and 25(OH)D-1α-hydroxylase activity. TLRs are a class of non-catalytic transmembrane pathogen-recognition receptors (PRRs) that interact with specific pathogen-associated molecular patterns (PAMPs) [95]. Calcitriol stimulates antimicrobial activities of macrophages and monocytes through VDR-RXR signaling, which triggers the production of cathelicidins that attach to microbial membranes to eliminate the bacteria and fungi [3,5,27,29,92,96,97,98]. Cathelicidin directly influences various respiratory viruses by disrupting viral envelopes [97,99,100,101]. This is also important in granulomatous inflammation such as TB, lymphomas, and sarcoidosis [3,79,102,103].

Many studies reported that low vitamin D levels are associated with an increased risk of infections and autoimmune diseases due to molecular mimicry [104,105]. Vitamin D can influence dendritic cell activity, inhibiting monocyte differentiation into dendritic cells and reducing IL-12 production [7,106,107,108]. 1,25-dihydroxivitamin D regulates NK cell activity, degranulation process, cytokine secretion, and TLR4 expression. This could demonstrate the benefit of vitamin D supplementation in patients with cancer [109]. Vitamin D regulates the intracellular TLRs differently, down-regulating TLR9, whereas TLR3 is unaffected. The decreased expression of TLR9 results in less IL-6 secreted. This highlights the association between vitamin D deficiency and the risk of developing autoimmune diseases [110].

Calcitriol intervenes in antigen-presenting cells (APC) differentiation and function by promoting APC to become more tolerogenic and decreasing the expression of major histocompatibility complex (MHC) class II and other similar molecules on the cell surface [111,112,113]. The data regarding whether calcitriol induces or inhibits NK cell function is uncertain [109,114,115].

In 1986 Rook and colleagues demonstrated that calcitriol inhibits the growth of Mycobacterium tuberculosis [116]. Liu P.T. and colleagues reported that TLR activation of human macrophages intervenes in killing intracellular Mycobacterium tuberculosis by stimulating the expression of VDR and vitamin D-1–hydroxylase genes. The authors also highlighted the role of TLRs and vitamin D in modulating innate immune responses. This link contributes to the differences found among human populations in synthesizing vitamin D and responding to microbial infection [96]. Other studies showed that calcitriol generates the expression of antimicrobial genes and the interaction between VDREs and containing promoter sequences of the cathelicidin antimicrobial peptide (camp) and defensin β2 (defB2) genes. Ligand binding results in VDR heterodimerization with DNA binding and retinoid X receptors to related VDREs composed of direct duplicates of consensus PuG(G/T)TCA motifs. This underlies the importance of new therapeutic strategies using calcitriol analogs in treating opportunistic infections [95,117,118]. Calcitriol and its analogs have the potential to induce the expression of camp gene in bronchial epithelial cells [119], keratinocytes [120], and myeloid cell lines [121]. 25(OH)D can also induce promoter sequences of the cathelicidin, underlining the antimicrobial function in cells that express 1α-hydroxylase encoded by the CYP27B1 allele [120]. CYP27B1 allele is expressed on various types of cells, and its function depends on the stimulation of each specific cell. Enhanced sensitivity to calcitriol is achieved through PAMPs, activating innate immune responses mediated via TLRs [95,122].

Activated human macrophages after being treated with mycobacterial 19 kDa lipoprotein, a TLR2 that interacts with specific PAMPs, demonstrated increased gene expression of both CYP27B1 and VDR, inducing promoter sequences of the cathelicidin through 25(OH)D and further bacterial killing [88,105,123,124]. A similar result regarding the innate immune response to infection was revealed by inducing cathelicidin expression in monocytes exposed to M. tuberculosis mediated through calcitriol. Calcitriol was able to induce bacterial killing through other factors such as nitric oxide synthase [74]. The expression of cathelicidin-small interfering RNA can enhance the pathway by which calcitriol interacts with the innate immune system. This underlies that cathelicidin is needed for calcitriol antimicrobial activity against M. tuberculosis [96].

Macrophages stimulated with bacterial lipopolysaccharide converted calcitriol to a more polar calcitriol-like metabolite [125]. Vitamin-D-mediated pathway regarding bacterial killing consists of the expression of cathelicidin that is suppressed by specific pathogens. In this matter, macrophages infected with Shigella suppress the expression of cathelicidin and defensin β2 to bypass innate antibacterial immunity [126]. Local induction of calcitriol synthesis can increase cathelicidin expression in promoting the antibacterial function. This mechanism is controlled by feedback pathways limiting a potential exaggerated inflammatory response that could arise from the activation of the immune system. Both down-regulation of TLR2 and TLR4 on monocytes and up-regulation of CD14 were inhibited by VDR antagonist ZK 159222, underlining that calcitriol requires VDR transcription factor activation on immunity receptors [127]. The reduced expression of TLR might inhibit inflammatory T lymphocyte responses that could generate T-helper 1 (Th1) lymphocyte autoimmunity. Feedback regulation of vitamin D is controlled by CYP24A1, the enzyme that catalyzes the synthesis of less active vitamin D metabolites [128].

Vitamin D and its metabolites intervene in vascular permeability and endothelial function through various genomic and non-genomic pathways. 25(OH)D and calcitriol stabilized vascular endothelium via a non-genomic pathway [129,130]. Calcitriol up-regulates endothelial nitric oxide synthase (eNOS), causing increased endothelial production of nitric oxide [131,132]. The increase in eNOS activity via calcitriol activation of VDR through intracellular adenylyl cyclase/cyclic adenosine monophosphate (AC/cAMP) and inositol trisphosphate/diacylglycerol (IP3/DAG) pathways leads to increased intracellular calcium level. VDR also triggers eNOS activation through the phosphoinositide 3-kinase/protein kinase b (PI3K/Akt) pathway, resulting in phosphorylation of serine-1779 on eNOS [133]. In an animal model, calcitriol stimulates endothelial-cadherin-based cellular junctions, inhibits stress fiber formation, inhibits the endothelial intracellular gaps organization, and limits endothelial damage in chronic kidney disease. Vitamin D and its metabolites prevent vascular dysfunction and local and systemic inflammation with tissue injury [134].

Calcitriol stimulates the expression of CYP24A1 in macrophages with no concomitant increase in CYP24A1 activity as calcitriol stimulates the expression of a splice variant form (CYP24A1-SV) that encodes a truncated amino-terminal protein [135]. The cytoplasm inactive state of CYP24A1-SV can limit vitamin D metabolism by confining the conversion to metabolites. The autocrine mechanism for bacterial killing is enhanced through the metabolism of vitamin D with PRR responses that interact with specific PAMPs. This mechanism is involved in macrophage-mediated immune responses, but also keratinocytes can induce cathelicidin through TLR2-mediated recognition of PAMPs in relation to the autocrine synthesis of calcitriol [136]. Keratinocytes can produce calcitriol in the presence of the transforming growth factor β1 and stimulate the production of cathelicidin mediated through TLR2 with the expression of CYP27B1 [136]. Interaction between transforming growth factor β1 and vitamin-D-mediated cathelicidin production involves wound repair through innate immune activation [136].

Vitamin D intervenes in gut integrity and maintaining a balanced relationship between host and gut microbiota. Vitamin D signaling maintains the integrity of intestinal epithelial cells and limits the bacterial lipopolysaccharide damage to intestinal epithelium [137,138]. Multiple studies have shown that vitamin D stimulates the expression of proteins that recognize intracellular pathogens [139,140] and promotes the production of antimicrobial proteins by the intestinal epithelial cells, intraepithelial lymphocytes, and Paneth cells [141,142]. These inhibit intestinal bacterial translocation and maintain intestinal homeostasis, which is thought to contribute to developing many auto-inflammatory and metabolic disorders.

## 3. Vitamin D Effects on the Adaptive Immune System

Vitamin D and VDR can influence B and T lymphocytes [6,27] (Figure 1). Several studies have reported that Vitamin D may intervene in B-cell differentiation and proliferation, decreasing antibodies and auto-antibodies synthesis with B-cell apoptosis. Vitamin D may also reduce T helper (Th) cell differentiation and proliferation and induce a more tolerogenic immune response than a pro-inflammatory status [143,144]. Vitamin D inhibits the synthesis of various pro-inflammatory Th1, Th9 and Th22 cytokines and stimulates the synthesis of anti-inflammatory Th2 cytokines. These findings could demonstrate the beneficial effect of vitamin D in minimizing the risk of developing autoimmune diseases [144].

Studies demonstrated that VDR expression in lymphocytes enhances vitamin D  as a pluripotent regulator of the innate immune system [75,145,146]. VDR expression is described in activated proliferating T lymphocytes [75] and B lymphocytes [147], implicating vitamin D as a modulator of the adaptive immune system. Inactive B lymphocytes do not present VDR; they upregulate VDR expression only when they are activated for proliferation through mitogens [77].

Initially, calcitriol suppression of immunoglobulin production was thought to be an indirect effect mediated via Th lymphocytes [148]. Further studies showed that calcitriol could directly influence B lymphocyte homeostasis [77]. In this concern, calcitriol can inhibit B cell differentiation into antibody-secreting plasma cells, inducing apoptosis, a process linked to DNA hypo-methylation [149,150]. This suggests the implication of vitamin D in B cell-related diseases such as systemic lupus erythematosus (SLE). Patients with SLE have significantly lower serum levels of calcitriol and 25(OH)D compared to controls [25,77].

As part of the adaptive immune system, Vitamin D intervenes in T lymphocyte proliferation and function [75]. Calcitriol targets Th lymphocytes by suppressing their proliferation and cytokine production, such as interleukin 2 (IL-2) [151]. The antigen-activated pluripotent Th0 lymphocytes generate various cytokines, including IL-2, IL-4, IL-10 and interferon γ (IFN-γ) [152]. Calcitriol directly inhibits [153] the expression of Th1 cytokines (IL-2, IFN-γ, tumor necrosis factor) [154] and stimulates Th2 (IL-3, IL-4, IL-5, IL-10) [153,154,155,156] or indirectly through APCs [113]. It also suppresses mature B cells to form plasma cells and class-switched memory B cells [77,157,158].

Calcitriol stimulates the expression of CTLA-4 and FoxP3, requiring the presence of IL-2 [159]. Calcitriol promotes Th1 cellular immunity more than Th2 humoral immunity. This is an essential key factor regarding the beneficial effects of vitamin D in autoimmune diseases [160]. Reports have shown that calcitriol stimulates the production of CD4+CD25+ Treg. Treg cells generate self-tolerance and contribute to preventing autoimmune disorders and graft-versus-host disease in transplantation [161].

Cytotoxic T lymphocytes (CTL), similar to Th cells, express both VDR and CYP27B1. Expression of VDR can appear in the presence of infection and mitogen stimulation, underlining a connection between the VDR signaling pathway and CTL activity [162,163]. Studies have reported that decreased CD4/CD8 ratio is associated with reduced levels of 25(OH)D [164]. Administration of 5000–10,000 IU of 25(OH)D increased CD4/CD8 ratio, suggesting immune suppression [165,166], but there is limited information about the direct effect of vitamin D on CTL. Induction and functions of CTL are influenced via direct activation of VDR and alteration in the expression of inflammatory cytokines through Th cells and APCs [143,163].

Calcitriol or its synthetic analogs can cause induction of Treg cells offering protection against autoimmune disorders and immune tolerance in organ transplantation [167,168]. Vitamin D targets for the adaptive immune system are the dendritic cells (DCs), especially a certain class of DCs known as myeloid DCs [169]. Calcitriol suppresses the maturation of DCs and the development of Th1 and promotes the induction of tolerogenic DCs and Treg [106,170]. A similar effect on DC could be obtained through CC-chemokine ligand (CCL) 22, which is a chemoattractant factor secreted by DCs that stimulates the synthesis of Treg. In myeloid DCs, calcitriol inhibits intracellular signaling nuclear factor κB [169].

## 4. Vitamin D Target Genes with Functions in the Immune System

**Figure 2:**
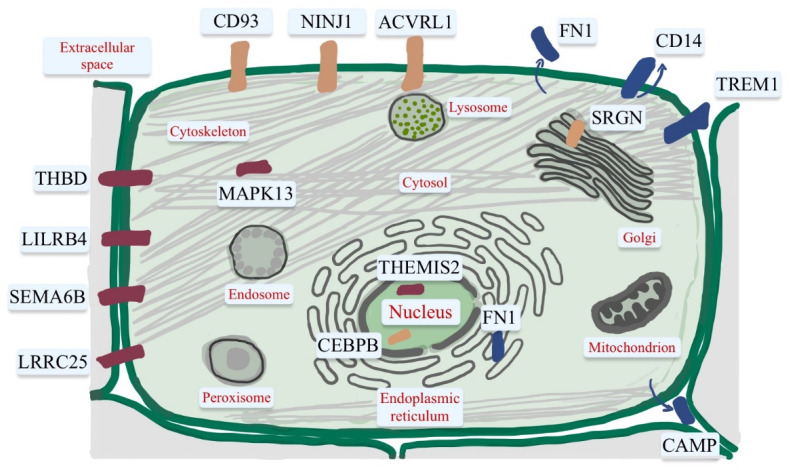
The proteins encoded by the 15 key genes are present at a certain location in the cell. The classification of the proteins is based on their transcriptome profile: orange—genes that encode proteins related to autoimmunity; purple—genes that encode proteins with a general function in infection; blue—proteins that are involved in acute response to infection.

Vitamin D target genes present a key role in the action of vitamin D in innate and adaptive immunity, and each function is demonstrated based on particular gene regulatory scenarios. A study described these genes by performing transcriptome-wide datasets based on peripheral blood mononuclear cells (PBMCs) and human monocytic cell line (THP-1), which were treated in vitro by calcitriol. These genes were described based on their VDR stimulation and their mRNA production. The following genes were identified in this study: ACVRL1, CAMP, CD14, CD93, CEBPB, FN1, MAPK13, NINJ1, LILRB4, LRRC25, SEMA6B, SRGN, THBD, THEMIS2 and TREM1. Vitamin D target genes were categorized based on their role in acute infection, infection in general and autoimmunity (Figure 2). These immune-related genes encoded proteins placed in the plasma membrane (ACVRL1, CD14, CD93, LILRB4, LRRC25, NINJ1, SEMA6B, THBD, TREM1) or are secreted (CAMP, FN1 and SRGN). In addition, the kinase MAPK13 is located in the cytoplasm, and the transcription factor CEBPB and the TLR scaffold protein THEMIS2 are placed in the nucleus [171].

CD14, TREM1, FN1, and CAMP are proteins involved in acute response to an infection via LPS/TLR4 signaling pathway [171]. The CD14 glycoprotein is mainly expressed in monocytes and macrophages and is located on the surface of the plasma membrane via glycosylphosphatidylinositol. CD 14 is a co-receptor for TLR 1-4, 6, 7 and 9 [172] and presents bacteria produced by LPS to TLR4 in inflammation [173]. Another vitamin D target gene located on monocytes and macrophages [174] is TREM1, associated with TLR4 signaling that generates inflammation in bacterial infection [175]. The extracellular matrix protein FN1 encoded by vitamin D, secreted by macrophages, epithelial cells and fibroblasts, is involved in inflammatory responses in LPS/TLR4 signaling, cell adhesion, and wound healing [176].

THBD, LILRB4, SEMA6B, LRRC25, MAPK13, and THEMIS2 genes encode proteins with a general function in infection [171]. THBD is secreted by monocytes, macrophages and endothelial cells [177] and reduces blood clots by turning thrombin pro-coagulative action to anti-coagulative. It also prevents pro-inflammatory responses of NF-kB signaling, binds to LPS and promotes LPS binding to CD14 and TLR4 [178]. The THBD gene is a vitamin D target in monocytes [179] and PBMCs [180]. LILRB4 is a receptor involved in the inhibition of APCs such as macrophages, monocytes, DCs and microglia, resulting in the production of TNF, bactericidal activity [181], and stimulation of Treg differentiation [182]. The LILRB4 gene is a vitamin D target gene in PBMCs [183]. One vitamin D target gene in bone is SEMA6B [184], belonging to the semaphorin protein family, which presents immune activity by controlling cell movements and cell-to-cell communication [185]. The LRRC25 protein acts on monocytes, granulocytes T cells, and DCs, inhibiting NF-kB signaling [186] and interferon [187]. It intervenes in response to viral infections by decreasing the production of inflammatory cytokines [188,189]. The kinase MAPK13 intervenes in LPS/TLR4 signaling and inflammatory responses along with MAPK11 [190]. It is a vitamin D target in skeletal muscle [191]. THEMIS2 is a TLR signal transduction modulatory protein that acts on macrophages, DCs, and B cells in TNF production induced by LPS [192] and T cell development [193].

CD93, NINJ1, CEBPB, ACVRL1, and SRGN genes encode proteins related to autoimmunity. CD93 glycoprotein acts on monocytes, endothelial cells, granulocytes, platelets and stem cells [194] and affects innate immunity in adhesion, phagocytosis and inflammation [195].    It functions as a DNA receptor for presenting to TLR9 and is involved in inflammatory responses in LPS/TLR4 signaling [196]. NINJ1 protein is expressed in myeloid and endothelial cells [197] and is described in the immune pathogenesis of multiple sclerosis [198] by promoting cell adhesion and leukocyte migration in inflammation of the endothelium. CEBPB gene is a vitamin D target in myeloid leukemia cells [199]. It proved to be involved in inflammation via Th17 in models of multiple sclerosis [200]. ACVRL1 is known to be a protein of the transforming growth factor-beta superfamily, which is associated with monocyte to macrophage differentiation [201], and with the signaling of the bone morphogenetic protein (BMP)9 and BMP10 [202]. SRGN is expressed by monocytes, macrophages, lymphocytes, mast cells, and endothelial cells [203]. It is involved in the secretion of inflammatory mediators via LPS [204].   

These 15 genes are considered the most important vitamin D targets in connection to immunity and biomarkers in diagnosing vitamin D deficiency related to immune diseases [171].

## 5. Vitamin D and Immune-Related and Autoimmune Diseases

The role of vitamin D supplementation in autoimmune diseases was studied based on the immunomodulatory effect of vitamin D. Animal experiments showed an essential role of calcitriol supplementation in autoimmune encephalomyelitis and collagen-induced arthritis, where vitamin D prevented the initiation and reduced disease progression [143,205,206]. Mouse models of enterocolitis presented a more severe disease course during vitamin D deficiency and decreased inflammation after vitamin D supplementation [207]. Despite various experimental studies, human clinical trials presented mixed results. Here we summarize the current findings in the literature regarding the role of vitamin D in immune-mediated and autoimmune diseases.

### 5.1. Psoriasis

Psoriasis is a chronic inflammatory disease, a hyper-proliferative disorder of the skin that affects 2–3% of the global population [208]. As 25(OH)D is mainly produced in the skin [209], and keratinocytes express VDR, calcitriol could be used to treat this disorder [210]. The inflammation generated in psoriasis after exposure to self-antigen is associated with Th1, Th17, and Th22 [211]. Calcitriol inhibits the differentiation of dendritic cells, antigen presentation, and the inflammatory activity of IL-1β, IL-6, IL-8, and TNF-α [212]. One study that analyzed if VDR gene polymorphisms are associated with psoriasis concluded that the rs7975232 allele was over-expressed in psoriasis patients compared to controls [213,214].

Even if it was initially described that cultured psoriatic fibroblasts were resistant to the anti-proliferative activity of calcitriol [215], other studies revealed that only approximately 20% of psoriatic patients presented this partial resistance [216]. A topical analog of calcitriol, calcipotriene (50 mcg/g), was developed to avoid a potentially hypercalcemic effect of calcitriol. Calcipotriene modulates the expression of pro-inflammatory cytokines (TNF-α, IFN-γ, IL-2, and IL-8) and is effective for treating psoriasis, and it does not generate significant hypercalcemia [217]. It was reported that skin irritation appeared mainly when applied to the face [218]. After it was described that calcitriol was effective at 15 mcg/g and did not cause skin irritation, topical treatment was developed as a 3 mcg/g formula for treating psoriasis that affects less than 10% of the body surface [210].

Studies described the association between vitamin D deficiency and patients with psoriasis [212,219], possibly because these patients avoid more exposure to sunlight. In this regard, various clinical trials have tried to find the proper vitamin D supplementation. The 25(OH)D level was inversely associated with all-cause mortality in patients with psoriasis [214,220]. A beneficial result in the PASI (psoriasis area and severity index) score and the serum level of 25(OH)D in patients who followed a six-month treatment with 60,000 IU of vitamin D2 administered every 2 weeks [221]. A similar result was described in a study that comprised psoriatic patients treated with daily 35,000 IUs of 25(OH)D for six months [222]. One study that included hospitalized patients during seven years of experience reported three patients with psoriasis that presented clinical skin improvement after using 20,000 to 50,000 IUs/day [223]. Another clinical trial reported no improvement in disease activity in patients with psoriasis after one year of monthly 100,000 IU of 25(OH)D [224]. The Endocrine Society clinical guideline recommends that patients with psoriasis should be tested and treated for vitamin D deficiency so that the 25(OH)D level would be 40–60 ng/mL (100–150 nmol/L) [38]. However, more extensive clinical trials are needed to adequately describe the efficacy of oral calcitriol psoriasis treatment.

### 5.2. Type 1 Diabetes

Type 1 diabetes (T1D) is encountered more frequently in countries located at higher latitudes with reduced sunlight exposure [225,226,227]. Areas such as Finland and Norway that are not exposed to sunlight for more than half a year have a high incidence of vitamin D deficiency [225,228]. The world’s highest incidence of T1D is described in Finland [229]. One meta-analysis involving 16 studies and more than 10,000 participants proved a significant inverse association between 25(OH)D concentration and the risk of developing T1D [214,230]. Other risk factors are proposed for the development of autoimmunity, such as exposure to toxic substances [231] and coxsackievirus B infections [232].

On the other hand, one mendelian randomization study including single nucleotide polymorphisms (SNPs) that are associated with 25(OH)D levels included in a large vitamin D genome-wide association study (GWAS) on more than 400,000 Europeans demonstrated that 25(OH)D levels were unlikely to present an impact on the risk of developing T1D [214,233]. Another case-control study did not register a significant association of each SNP (DHCR7, GC, CYP2R1 and CYP24A1 genes) with T1D but demonstrated a combined effect of multiple risk alleles [214,234].

The mechanisms involved in T1D comprise the generation of autoantibody and autoreactive Th1 and CTL, resulting in immune-mediated destruction of insulin-producing pancreatic β cells [235,236]. In animal experiments, calcitriol administration led to Treg stimulation and Th1 inhibition, reducing the incidence of T1D [168]. Vitamin D can be protective in developing T1D by stimulating insulin secretion in the pancreatic β cells via direct binding with VDR [225]. Some studies report a reduced risk of T1D in children with a higher vitamin D intake [237]. A clinical trial developed in Finland described a reduction of 88% in the risk of developing T1D in children who were given daily 2000 IU of 25(OH)D during their first year of life [238]. The EURODIAB multicenter case-control study reported a reduced risk for T1D if vitamin D was supplemented in any dose [237]. Other trials revealed that vitamin D supplementation managed to promote the reduction of the daily insulin dose and increase C-peptide levels [239,240,241]. Vitamin D intake with a normal 25(OH)D level is important in early childhood as it offers protection in the development of T1D. Vitamin D supplementation can improve the disease activity but does not influence morbidities and mortalities.

### 5.3. Multiple Sclerosis

Multiple sclerosis (MS) is encountered more frequently in countries located at a higher latitude with reduced exposure to sunlight, similar to type 1 diabetes [200,227]. Studies showed that a decrease of 41% in the risk of MS was obtained with every 20 ng/mL (50 nmol/L) increase in serum 25(OH)D levels above 24 ng/mL (60 nmol/L) [242]. The same study reported that women who had an intake of daily vitamin D higher than 400 IU presented a 41% decreased risk of developing MS [242].

A Mendelian Randomization (MR) study using information regarding MS and 25(OH)D levels from large European populations described that a decrease in genetically lowered 25(OH)D level by one standard deviation (SD) resulted in a 2-fold increase in developing MS [243]. Another MR analysis using genome-wide 25(OH)D-associated SNPs from a meta-analysis conducted in 401,460 British UK Biobank participants and 42,274 samples of European ancestry. Increased 25(OH)D concentration is associated with a decreased risk of developing MS. The authors found limited evidence supporting the protective effect of 25(OH)D on obesity, breast and prostate cancer, and autoimmune, metabolic, cardiovascular, and neuro-psychiatric diseases [244]. The largest GWAS for serum 25(OH)D includes 401,460 participants and 14,498 MS cases and 24,091 controls of European ancestry. A causal association was established with the increase of 25(OH)D based on genetic changes, resulting in a decreased MS risk [245]. Another 2-sample MR determined the genetic causal effect of vitamin 25(OH)D and concluded that each increase in the natural-log-transformed vitamin D concentration resulted in a 43% decrease in the odds ratio of MS (OR 0.57, 95% CI 0.41–0.81, *p* = 0.001) [61,246].

Vitamin D deficiency in MS stimulates auto-inflammation of the central nervous system through the dysregulation of Th cells, CTL, B cells, and NK cells, damaging neurons and oligodendrocytes [247,248]. There is an individual predisposition for developing MS, such as HLA-DRB1*1501 [203]. Vitamin D response elements are also situated in the promoter region of the HLA-DRB1 gene [249,250,251]. Among gene variants that are associated with developing MS are CYP27B1 and CYP24A1, alleles that are involved in the vitamin D pathway [214,252]. Various immune actions of calcitriol are similar to those of interferon-beta, an MS immunomodulatory treatment. There are conflicting results based on the therapeutic role of vitamin D in MS. Some studies revealed improvement in the relapse rate and MRI findings on an increased dose of vitamin D (14,000 IU/day) [253].

The treatment dosages of vitamin D vary significantly among various studies. They can comprise high doses of vitamin D of 50,000 IU/day or 1000 IUs/kg/day to obtain an increase in serum 25(OH)D up to 200–300 ng/mL (500–750 nmol/L). This high vitamin D supplementation improved symptoms and MRI findings in MS patients who did not respond to or refused conventional MS treatment [224]. One study described the treatment applied to 319 patients with a broad spectrum of autoimmune diseases (MS and non-MS such as vitiligo, psoriasis, inflammatory bowel disease, RA) that were treated for more than 3.5 years with high doses of orally 25(OH)D up to 1000 IU/kg. The study demonstrated that these doses were safe regarding calcium metabolism and renal function when patients followed a strict diet and fluid recommendations [254]. There are no RCTs to support the safety of the high dose of vitamin D (1000 IU/kg/day). Still, supplementation with lower doses of vitamin D (up to 14,000 IUs/day) proved to control the disease activity.

### 5.4. Inflammatory Bowel Diseases

The risk of inflammatory bowel disease (IBD) is more common in countries located at a higher latitude with reduced exposure to sunlight, similar to T1D and MS [227,255]. IBD presents a defective innate and adaptive immunity, with increased intestinal permeability, and imbalanced gut microbiota, resulting in chronic inflammation of the intestine [256,257,258].   

Vitamin D deficiency in IBD is caused by micelles and chylomicron that cannot form and absorb intestinal vitamin D [259,260], resulting in an increased risk of osteomalacia and osteoporosis [259,260,261]. Therefore, these patients should be tested and treated for vitamin D deficiency with an increased dose of vitamin D so that the 25(OH)D level would be at least 30 ng/mL (75 nmol/L) [38]. The decreased 25(OH)D level is a biomarker for IBD activity and a predictor of poor prognosis in these patients [214,262].

The inflammatory response in CD comprises Th1 stimulation, while in UC, Th2 is promoted [256,263], and Th17 is involved in both diseases [256,264,265]. Studies reported that calcitriol stimulates Treg and inhibits Th1 and Th17 activity. It also intervenes on intestinal mucosal cells by stimulating proteins involved in membrane junction integrity and intracellular pathogen recognition. Vitamin D is essential in stimulating the production of antibacterial proteins such as cathelicidin, angiogenin, and defensin [139,140,262,265,266].

A prospective study from the Nurses’ Health Study revealed that a high 25(OH)D level was connected with a decrease of 46% in developing Crohn’s disease (CD) and 35% in developing ulcerative colitis (UC) [267]. Various clinical trials reported that vitamin D supplementation was beneficial in patients with IBD by decreasing relapse rate [268] and improving bacterial intestinal homeostasis in patients with CD [269]. One study based on healthy individuals with vitamin D deficiency who received daily doses of either 600, 4000, or 10,000 IU for 6 months presented improved immune response with increased expression of Treg and improvement of gut microbiota [270]. Unable to absorb vitamin D, patients with IBD need higher doses of vitamin D to achieve normal serum 25(OH)D levels and help prevent the development of bone fragility, osteoporosis, and osteomalacia.

### 5.5. Rheumatoid Arthritis

The risk of rheumatoid arthritis (RA) is associated with low levels of 25(OH)D [271]. Studies reported that women with higher doses of vitamin D presented a 33% reduced risk for developing RA [271], and lower serum 25(OH)D levels were associated with disease activity [271,272]. Although these patients have reduced exposure to sunlight because of their disease, treatment with vitamin D is considered beneficial due to Th1 and Th17 suppression and Treg activity stimulation [273]. These immunologic responses promote chronic synovial inflammation [274,275].

There are mixed results reported from studies using adjunctive treatment with vitamin D for RA. Clinical trials described important pain relief in RA patients that received 500 IU daily of 25(OH)D with disease-modifying anti-rheumatic drugs (DMARDs) and calcium compared to those that did not receive vitamin D supplementation [276].

Another study presented RA patients receiving either 22-oxa-1,25-dihydroxyvitamin D or calcitriol or placebo, which led to decreased swollen joints and improved disease activity scores in the group of patients that received 22-oxa-1,25-dihydroxyvitamin D and those with calcitriol compared with the placebo group [277]. Other clinical trials that used vitamin D2 [278], 25(OH)D [279,280], or 1α-hydroxyvitamin D3 [281] presented less convincing results in the treatment of RA patients. One meta-analysis identified 9 RCTs of vitamin D treatment for at least 3 months in autoimmune diseases, which included five studies of RA patients, but with no statistical significance. These patients presented a decrease in the number of flares, patient global visual analog scale score (VAS) and disease activity score 28 (DAS-28) with vitamin D supplementation [214,266,282]. Vitamin D intake associated with serum 25(OH)D levels of 40–60 ng/mL (100–150 nmol/L) may result in a lower risk of RA, but there is still scarce evidence on this subject.

### 5.6. Systemic Lupus Erythematosus

Systemic lupus erythematosus (SLE) is a chronic, multisystem, autoimmune disease that mostly appears in young women. Studies described that SLE is associated with low vitamin D levels in disease expression, relapses, and pathogenesis [214,283,284,285,286,287,288,289,290,291,292]. In SLE, there is no association between vitamin D and pro-inflammatory cytokines. Most studies describe that low vitamin D correlates with disease activity, but two studies reported that vitamin D did not influence disease activity [293]. Vitamin D deficiency was more prevalent in unsupplemented SLE participants living at a latitude beyond the 37˚ parallel north [294]. Some additional risk factors for vitamin D insufficiency are described in SLE patients, such as reduced sun exposure, renal disease, VDR genetic polymorphisms with decreased cell responsiveness to the hormone and the genetic variants that encode important enzyme regulators of endogenous production [295,296].

Moreover, vitamin D has a protective role in lupus nephritis induced by autoantibodies [297]. Reduced vitamin D concentration is associated with worse disease activity, fatigue [298], cardiovascular disease [299], and cognitive impairment [300,301]. One study demonstrated that vitamin D deficiency is associated with more active disease at first admission and over time, and more severe lupus flares [285,302].

There is also a high prevalence of vitamin D deficiency in newly diagnosed SLE patients and a significant association between vitamin D level and the decreased C3 level [214,303]. Low 25(OH)D concentration is associated with increased levels of memory B cells in well-controlled SLE patients [214,304] and an increased number of Treg cells [305].

Vitamin D status and CYP24A1 might contribute to the predisposition of SLE in individuals with genetic risk for SLE [306]. Pregnant women with SLE and low vitamin D presented higher morbidity [288]. Vitamin D deficiency often correlates with low bone mineral density (BMD) [287]. The presence of FokI and TaqI variants was demonstrated in an eastern Indian cohort of patients with SLE [307]. Even if vitamin D is associated with disease activity in SLE, further recommendations are needed regarding vitamin D target dose and target levels [7].

Vitamin D supplementation should be part of SLE treatment [294] to reduce disease SLE activity as recommended by two randomized controlled trials [308,309], one open clinical trial [310] and a cohort study [311]. On the other hand, two cohort studies [251,312] and an RCT [313] failed to describe any significant variation.

Vitamin D supplementation can also be used to prevent glucocorticoid-induced osteoporosis [314]. Current vitamin D supplementation strategies do not focus on individual recommendations and can be insufficient in raising vitamin D concentrations in every patient. In this regard, a treat-to-target approach could be more suitable. For a better disease approach, an initial measurement of vitamin D level should be performed for each patient. 

### 5.7. Systemic Sclerosis

Systemic sclerosis (SSc) is an autoimmune multi-organ disease characterized by vasculopathy and fibrosis involving the skin and/or lungs, heart and kidneys [315,316,317]. Multiple studies described lower vitamin D levels in SSc patients [318,319]. Still, clinical manifestations were not connected to the degree of vitamin D deficiency [320]. Neither was age, gender, disease activity with digital ulceration or systemic involvement, nor the type of auto-antibodies. Still, the degree of vitamin D deficiency had an inverse correlation with skin sclerosis [321].   

Low vitamin D levels in SSc can be explained by reduced sunlight exposure, inadequate epidermal production of vitamin D in the epidermis due to skin fibrosis, and diminished gastrointestinal malabsorption [322]. Another reason for low levels of vitamin D could be the presence of antivitamin D antibodies, as shown in a study that demonstrated that the antivitamin D antibodies were found in 87% of SSc patients and 42% of controls [323].

Regarding the SSc pathogenesis, the transition from fibroblast to activated myofibroblast is an important event in SSc involving TGF-β signaling, epigenetic mechanisms and various other cellular pathways [324]. Vitamin D effect on fibrosis in SSc concerns impaired VDR signaling with reduced expression resulting in hyperactive TGF-β signaling and abnormal fibroblast activation [325]. When analyzing vitamin D and two major regulators, SSc patients presented a reduced FGF23/α-Klotho index and its log10 related to disease activity score [326].

The studies regarding the impact of vitamin D deficiency and clinical features and disease activity of SSc are controversial because of the small sample size, SSc being a rare disease [327]. A study that included 327 SSc patients described an inverse relationship between vitamin D concentration and skin involvement [322]. A cohort study reported that pulmonary involvement was more often encountered in patients with vitamin D deficiency than controls, but with no statistical significance [328]. Some studies suggested an association between vitamin D level and organ involvement, particularly pulmonary and cardiac [329], and pulmonary artery hypertension [330,331]. Further research is required to understand the role of vitamin D in SSc and whether its supplementation could impact disease activity or influence disease onset.

### 5.8. Miscellanea

Antiphospholipid syndrome is a multisystem autoimmune disease characterized by hypercoagulability, which may be associated with recurrent fetal loss and thromboembolic events in the presence of elevated titers of antiphospholipid antibodies (aPL) [332,333]. Studies described that low vitamin D levels are associated with antiphospholipid syndrome [334,335,336,337] and vitamin D deficiency with clinically-defined thrombotic events [338].

Immunoglobulin A nephropathy (IgAN) is the most frequent primary form of glomerulopathy encountered in the western world. It is a disease that involves genetic and autoimmune mechanisms and environmental factors. It is a significant cause of renal impairment, which requires proper treatment to avoid irreversible damage [339]. Studies have shown an association between vitamin D and IgAN. Vitamin D deficiency resulted in poorer clinical outcomes and more severe renal disease [340].

In autoimmune hepatitis (AIH), some of the VDR and CTLA4 variants are involved in triggering the immune process, these genotype variants being linked with fatty acid synthase (FAS) promoter variants or pro-inflammatory cytokines that are part of the TNF superfamily. This mechanism holds great promise for discovering disease-specific liver fibrosis [341,342,343,344].

Low vitamin D levels were correlated in patients with Sjögren syndrome (SS). Vitamin D may intervene in SS pathogenesis, influencing the presence of extra-glandular symptoms such as lymphoma or neuropathy [345]. One meta-analysis, including 18 studies with SS patients, concluded that patients with vitamin D deficiency associated higher severity scores of dry eye symptoms, such as lower Schirmer’s test scores and higher ocular surface disease index (OSDI) scores [214,346].

Moreover, vitamin D was also correlated with immune thrombocytopenic purpura, chronic idiopathic neutropenia, autoimmune hemolytic anemia, and Evans’ syndrome. Vitamin D deficiency can influence disease severity in autoimmune cytopenias, underlining the protective role in the development of autoimmunity [319].

## 6. Conclusions

In summary, vitamin D is essential in calcium, phosphate, and bone homeostasis and acts as an immunomodulator, influencing the innate and adaptive immune systems to fight against pathogens. Clinical trials underline the significance of maintaining vitamin D status within the normal range because a low serum level of 25(OH)D is correlated with various immune-related disorders, including autoimmune diseases. There is increased evidence that vitamin D supplementation could improve the treatment of autoimmune disorders. Another question is whether vitamin D is beneficial as an immunomodulatory agent in most diseases based on mixed findings from clinical trials. Healthcare institutions should develop programs to educate the public about the role of vitamin D. Food should be fortified with vitamin D to reduce the risk of vitamin D deficiency at risk ages for developing autoimmune disorders in childhood, in young and middle-aged adults, and during pregnancy. For vitamin D to have immunomodulatory functions, it is probably required the production of calcitriol in monocytes and macrophages. Further questions still need to be answered to fully understand vitamin D’s complete effect on the immune system.

## Data Availability

Not applicable.
